# Supplementary figures and images for: Human umbilical cord-derived mesenchymal stem cells alleviate autoimmune hepatitis by inhibiting hepatic ferroptosis
Source: PLoS One. 2025 Dec 4;20(12):e0337060. doi: 10.1371/journal.pone.0337060 (PMC12677442; doi:10.1371/journal.pone.0337060)

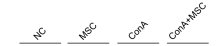

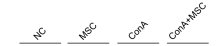
Figure 7B


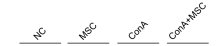

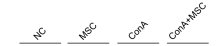

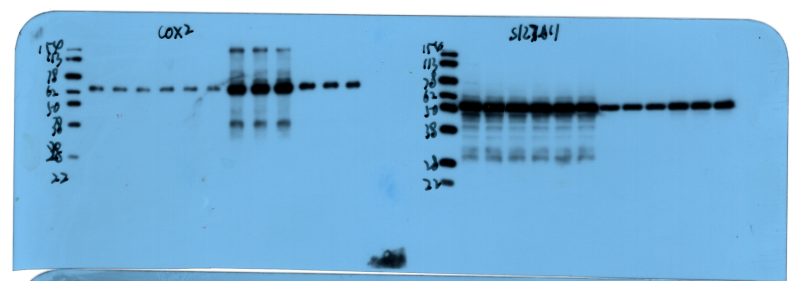


- 62 kDa

-50 kDa


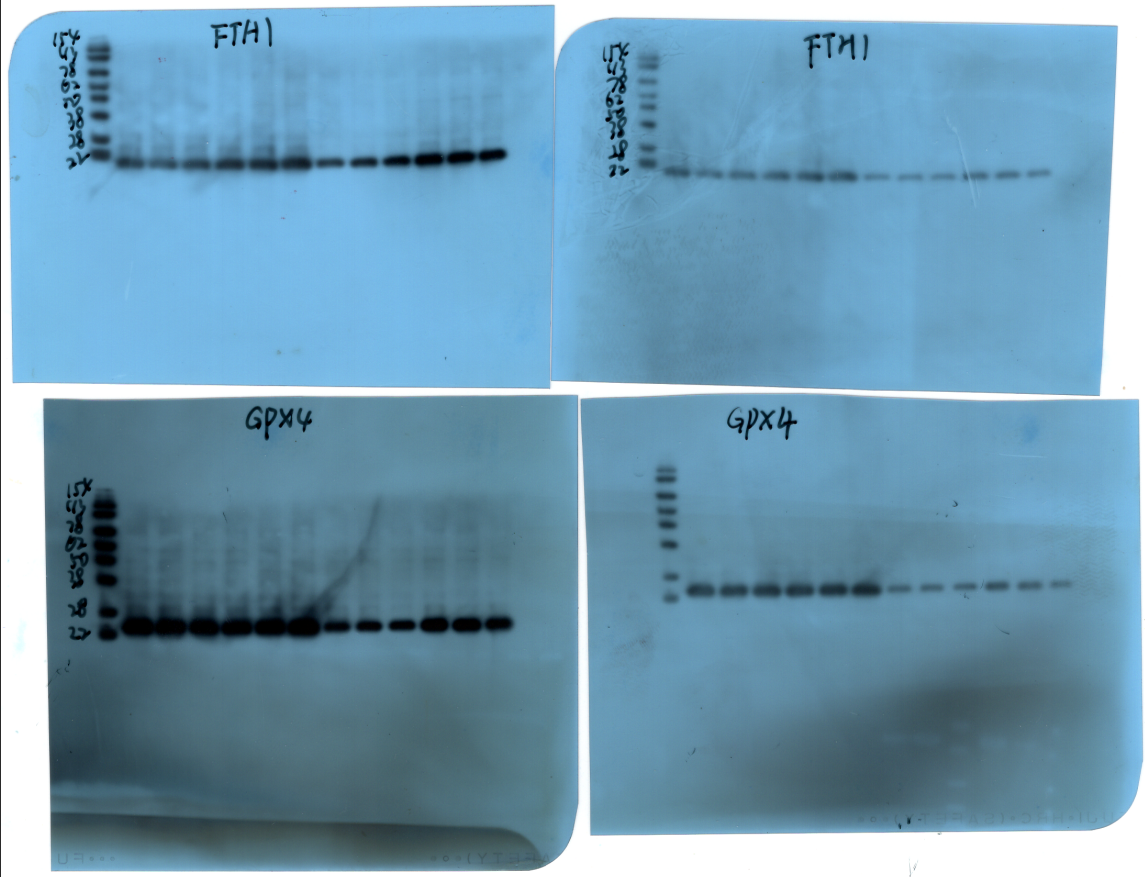

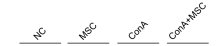

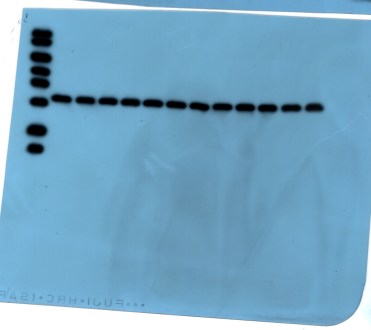


- 22 kDa

- 22 kDa

- 38 kDa

β-Actin

Supplement: S1 File — (DOCX) [file pone.0337060.s003.docx]

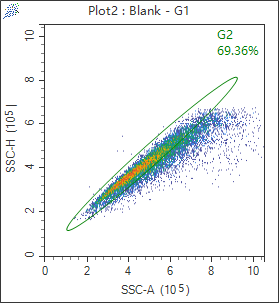

Supplement: S3 File — (ZIP) [file pone.0337060.s005.zip › Data/MSC Flow Cytometric Characterization Identification/Figure/20250811-MSC-Blank-Plot2.jpg]

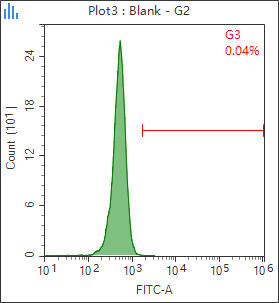

Supplement: S3 File — (ZIP) [file pone.0337060.s005.zip › Data/MSC Flow Cytometric Characterization Identification/Figure/20250811-MSC-Blank-Plot3.jpg]

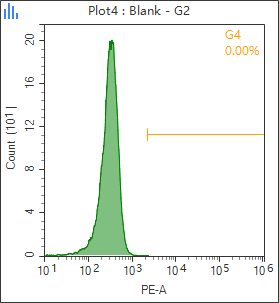

Supplement: S3 File — (ZIP) [file pone.0337060.s005.zip › Data/MSC Flow Cytometric Characterization Identification/Figure/20250811-MSC-Blank-Plot4.jpg]

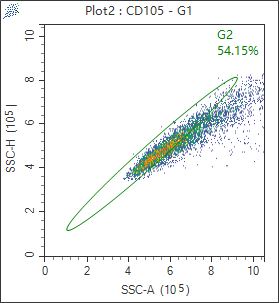

Supplement: S3 File — (ZIP) [file pone.0337060.s005.zip › Data/MSC Flow Cytometric Characterization Identification/Figure/20250811-MSC-CD105-Plot2.jpg]

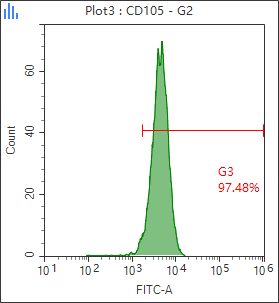

Supplement: S3 File — (ZIP) [file pone.0337060.s005.zip › Data/MSC Flow Cytometric Characterization Identification/Figure/20250811-MSC-CD105-Plot3.jpg]

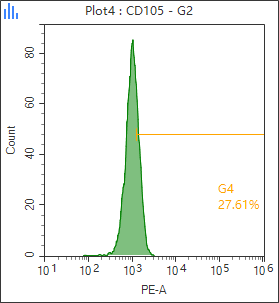

Supplement: S3 File — (ZIP) [file pone.0337060.s005.zip › Data/MSC Flow Cytometric Characterization Identification/Figure/20250811-MSC-CD105-Plot4.jpg]

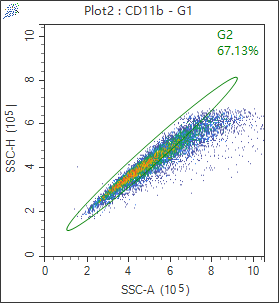

Supplement: S3 File — (ZIP) [file pone.0337060.s005.zip › Data/MSC Flow Cytometric Characterization Identification/Figure/20250811-MSC-CD11b-Plot2.jpg]

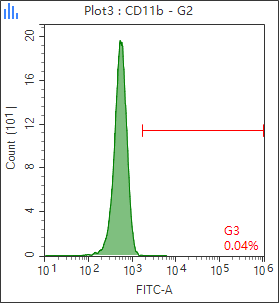

Supplement: S3 File — (ZIP) [file pone.0337060.s005.zip › Data/MSC Flow Cytometric Characterization Identification/Figure/20250811-MSC-CD11b-Plot3.jpg]

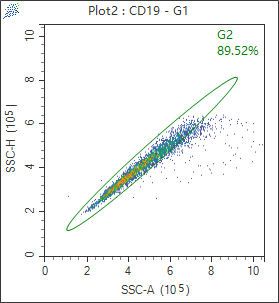

Supplement: S3 File — (ZIP) [file pone.0337060.s005.zip › Data/MSC Flow Cytometric Characterization Identification/Figure/20250811-MSC-CD19-Plot2.jpg]

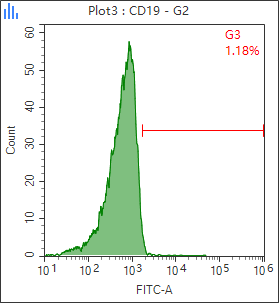

Supplement: S3 File — (ZIP) [file pone.0337060.s005.zip › Data/MSC Flow Cytometric Characterization Identification/Figure/20250811-MSC-CD19-Plot3.jpg]

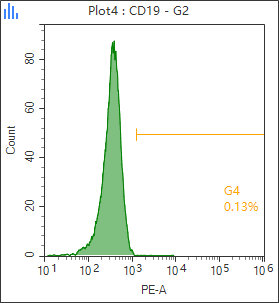

Supplement: S3 File — (ZIP) [file pone.0337060.s005.zip › Data/MSC Flow Cytometric Characterization Identification/Figure/20250811-MSC-CD19-Plot4.jpg]

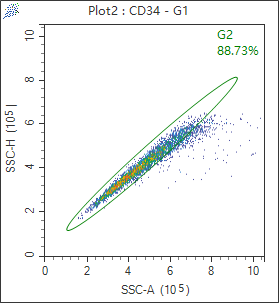

Supplement: S3 File — (ZIP) [file pone.0337060.s005.zip › Data/MSC Flow Cytometric Characterization Identification/Figure/20250811-MSC-CD34-Plot2.jpg]

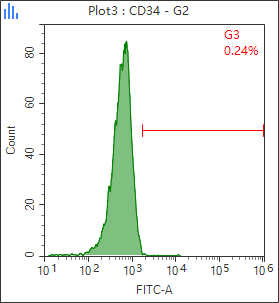

Supplement: S3 File — (ZIP) [file pone.0337060.s005.zip › Data/MSC Flow Cytometric Characterization Identification/Figure/20250811-MSC-CD34-Plot3.jpg]

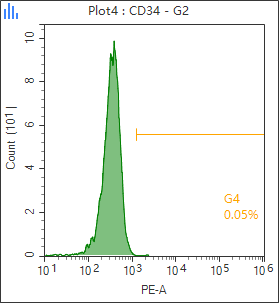

Supplement: S3 File — (ZIP) [file pone.0337060.s005.zip › Data/MSC Flow Cytometric Characterization Identification/Figure/20250811-MSC-CD34-Plot4.jpg]

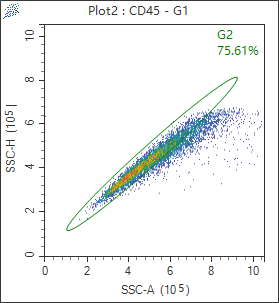

Supplement: S3 File — (ZIP) [file pone.0337060.s005.zip › Data/MSC Flow Cytometric Characterization Identification/Figure/20250811-MSC-CD45-Plot2.jpg]

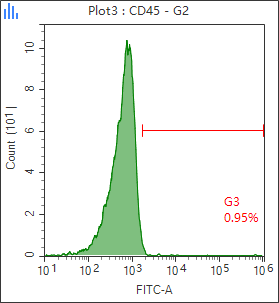

Supplement: S3 File — (ZIP) [file pone.0337060.s005.zip › Data/MSC Flow Cytometric Characterization Identification/Figure/20250811-MSC-CD45-Plot3.jpg]

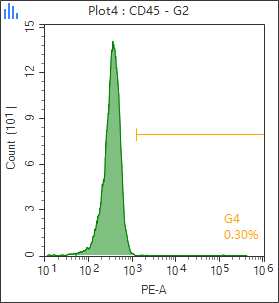

Supplement: S3 File — (ZIP) [file pone.0337060.s005.zip › Data/MSC Flow Cytometric Characterization Identification/Figure/20250811-MSC-CD45-Plot4.jpg]

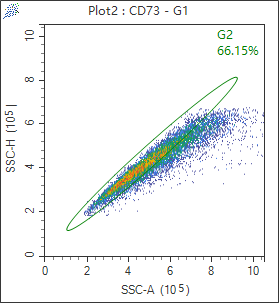

Supplement: S3 File — (ZIP) [file pone.0337060.s005.zip › Data/MSC Flow Cytometric Characterization Identification/Figure/20250811-MSC-CD73-Plot2.jpg]

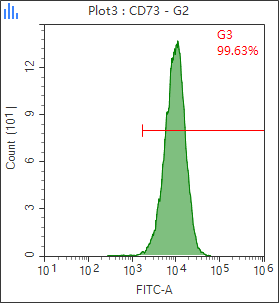

Supplement: S3 File — (ZIP) [file pone.0337060.s005.zip › Data/MSC Flow Cytometric Characterization Identification/Figure/20250811-MSC-CD73-Plot3.jpg]

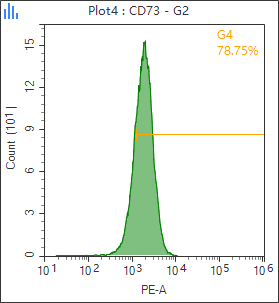

Supplement: S3 File — (ZIP) [file pone.0337060.s005.zip › Data/MSC Flow Cytometric Characterization Identification/Figure/20250811-MSC-CD73-Plot4.jpg]

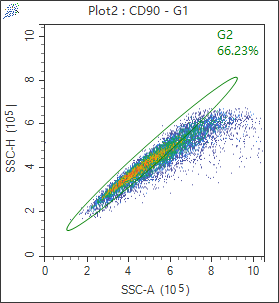

Supplement: S3 File — (ZIP) [file pone.0337060.s005.zip › Data/MSC Flow Cytometric Characterization Identification/Figure/20250811-MSC-CD90-Plot2.jpg]

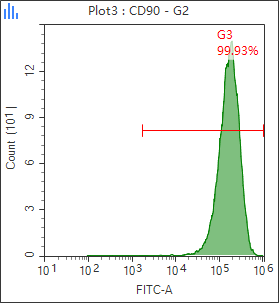

Supplement: S3 File — (ZIP) [file pone.0337060.s005.zip › Data/MSC Flow Cytometric Characterization Identification/Figure/20250811-MSC-CD90-Plot3.jpg]

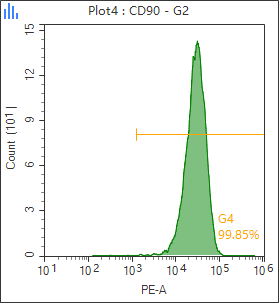

Supplement: S3 File — (ZIP) [file pone.0337060.s005.zip › Data/MSC Flow Cytometric Characterization Identification/Figure/20250811-MSC-CD90-Plot4.jpg]

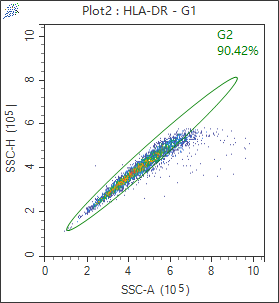

Supplement: S3 File — (ZIP) [file pone.0337060.s005.zip › Data/MSC Flow Cytometric Characterization Identification/Figure/20250811-MSC-HLA-DR-Plot2.jpg]

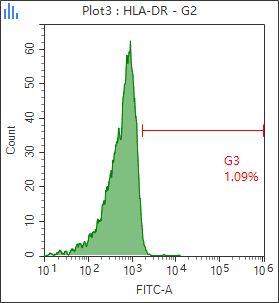

Supplement: S3 File — (ZIP) [file pone.0337060.s005.zip › Data/MSC Flow Cytometric Characterization Identification/Figure/20250811-MSC-HLA-DR-Plot3.jpg]

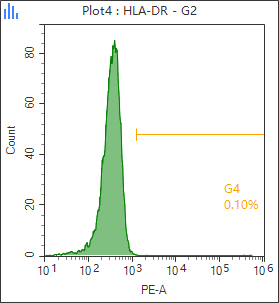

Supplement: S3 File — (ZIP) [file pone.0337060.s005.zip › Data/MSC Flow Cytometric Characterization Identification/Figure/20250811-MSC-HLA-DR-Plot4.jpg]
