# Supplementary material for: Human umbilical cord-derived mesenchymal stem cells alleviate autoimmune hepatitis by inhibiting hepatic ferroptosis
Source: PLoS One. 2025 Dec 4;20(12):e0337060. doi: 10.1371/journal.pone.0337060 (PMC12677442; doi:10.1371/journal.pone.0337060)
Supplement: S2 File — (DOCX) [file pone.0337060.s004.docx]

Figure S2

NC

MSC

ConA

ConA+MSC


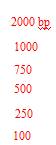

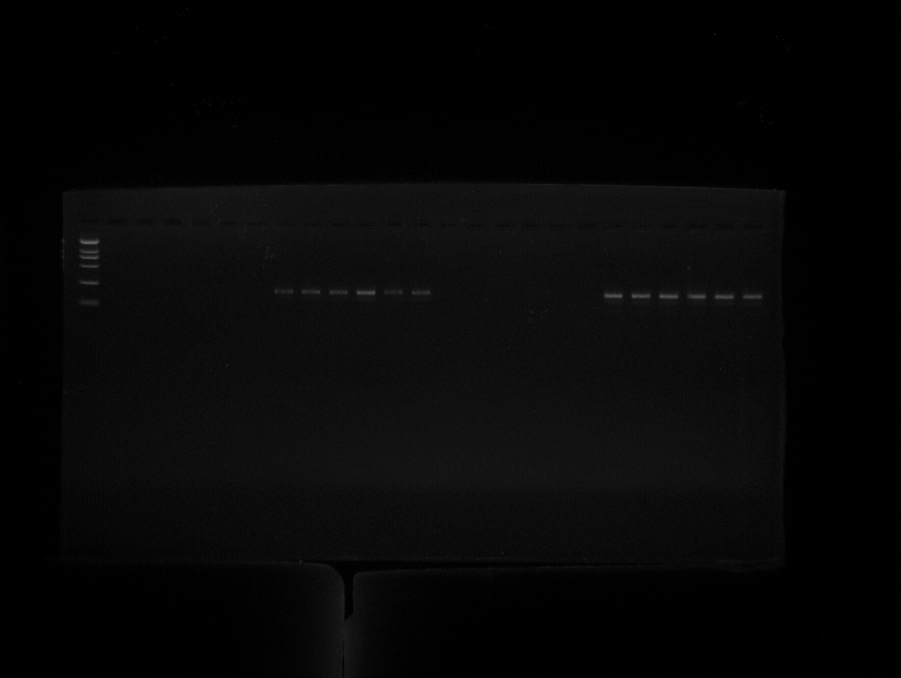


Zs-Green

MSC

NC

ConA


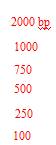

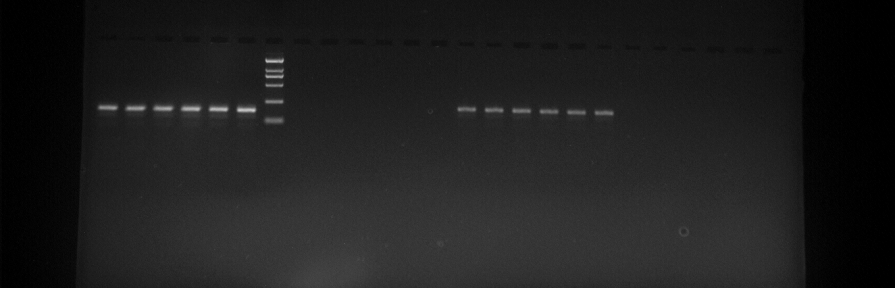


Luciferase

ConA+MSC


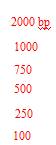

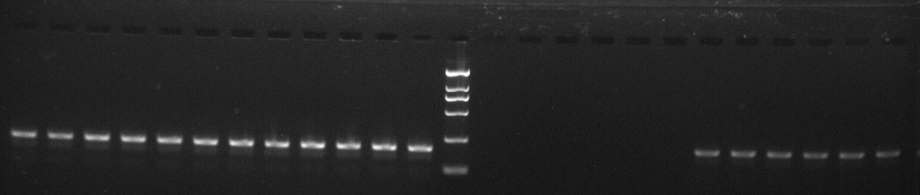


Luciferase

NC


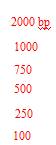

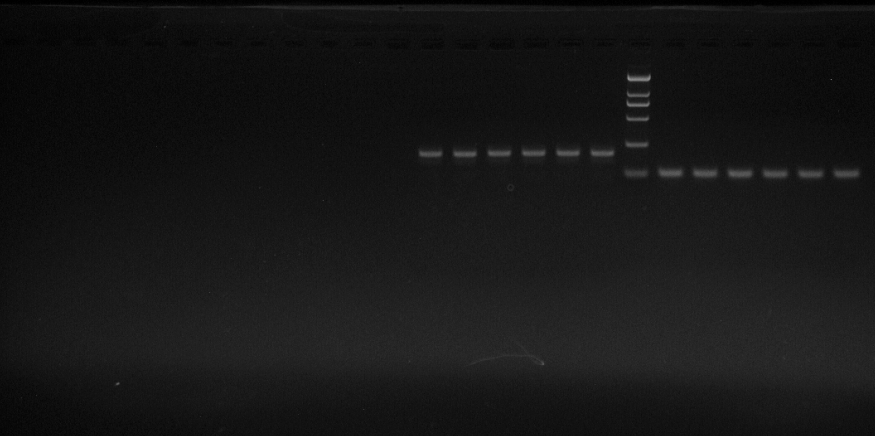


18S


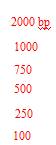

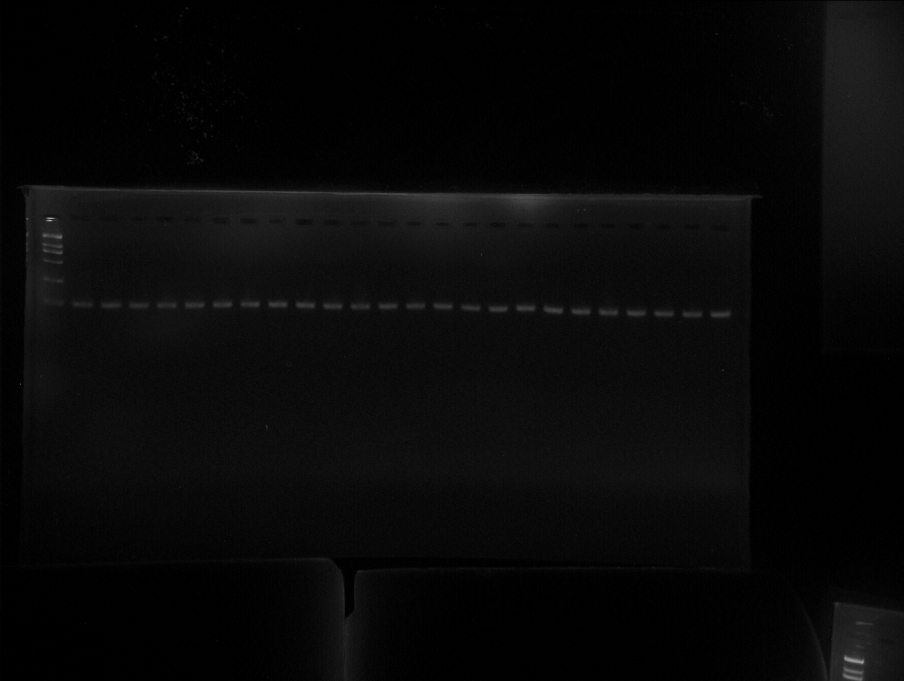


18S

MSC

ConA+MSC

ConA
