# Supplementary material for: Human umbilical cord-derived mesenchymal stem cells alleviate autoimmune hepatitis by inhibiting hepatic ferroptosis
Source: PLoS One. 2025 Dec 4;20(12):e0337060. doi: 10.1371/journal.pone.0337060 (PMC12677442; doi:10.1371/journal.pone.0337060)

Countess™ 3 Report

August 06, 2022 09:43

Summary

Sample name: MSC
Count ID: 1082

Results

Trypan Blue corrected

	Concentration	
Total		2.67 x 10^6/mL
Live	99%	2.65 x 10^6/mL
Dead	1%	2.09 x 10^4/mL

Settings

	Live	Dead	
Acquisition			
Intensity	64	64	
Focus	64	64	
Gating			
Protocol	lps		
Size	2, 45	0, 70	
Brightness	0, 255	0, 255	
Circularity	0, 100	0, 100	

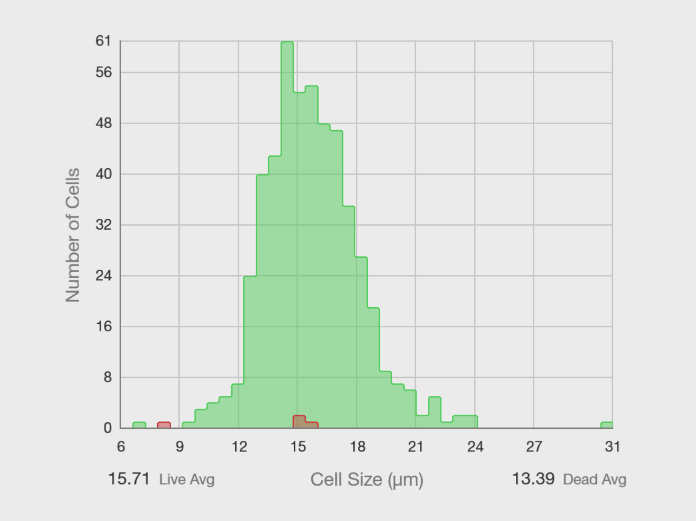

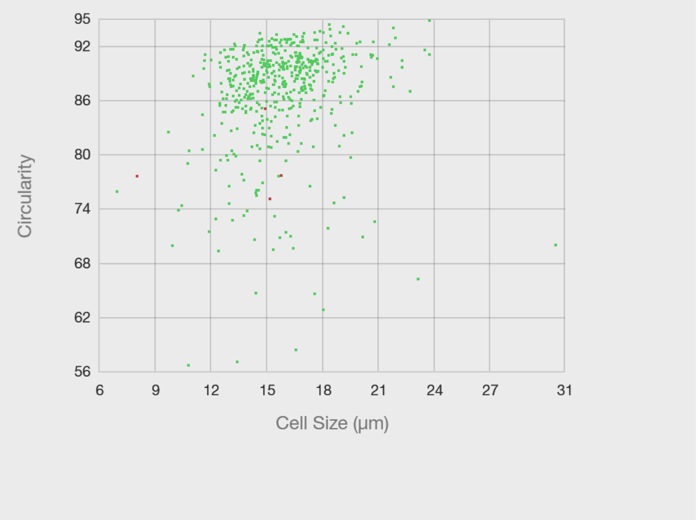

Countess™ 3 FL

Automated Cell Counter

invitrogen
by Thermo Fisher Scientific

Countess™ 3 Report

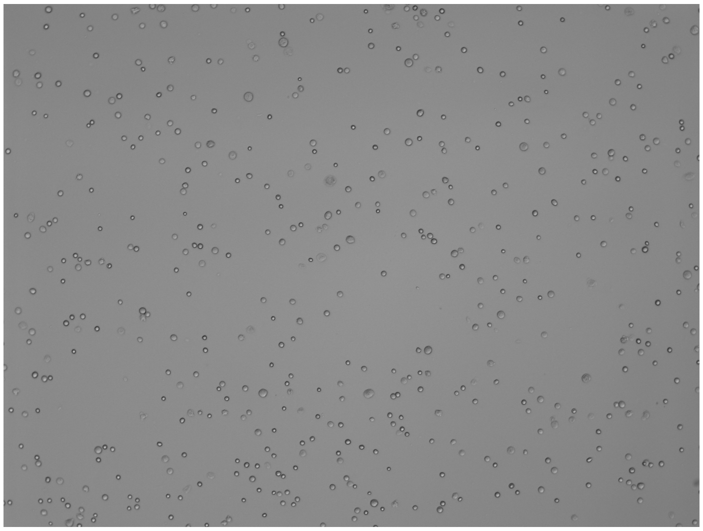

Supplement: S3 File — (ZIP) [file pone.0337060.s005.zip › Data/MSC_Report.pdf]
