# Supplementary material for: Human umbilical cord-derived mesenchymal stem cells alleviate autoimmune hepatitis by inhibiting hepatic ferroptosis
Source: PLoS One. 2025 Dec 4;20(12):e0337060. doi: 10.1371/journal.pone.0337060 (PMC12677442; doi:10.1371/journal.pone.0337060)
Supplement: Attachment — Submitted filename: response to reviewers comments.docx [file pone.0337060.s007.docx]

**Comments to the Author**

**Reviewer #1: Page14/line5: `randomly assigned into four groups (n = 3 per group), the number in each group is too small and impossible to run the statistics. Please add more animals in each group and reevaluate the experiment.**

Response: We appreciate the reviewer’s concern. We repeated the experiments in an additional six mice and incorporated the new data into the revised figures. One important clarification regarding the repeated experiments is that the two GLU measurements were performed using different assay kits. The original data were obtained with the MEIMIAN Glutamic Acid (Glu) Assay Kit (Cat. No. ADS-F-AJS007), whereas the repeat experiments used the Elabscience Glu Colorimetric Assay Kit (Cat. No. E-BC-K903-M). Owing to differences in the calculation methodologies between the two kits, the absolute values varied. Importantly, however, the intergroup trends remained consistent across both experiments.

**Page14/line8-9: how do you determine the cell number to inject. 1x10^6 cells per animal is too big amount. Is there any scientific reasons or references to support choosing this number. Please provide BW and gender in each group.**

**After cell injection, how many hUC-MSCs were reached and retained in the liver.**

Response: The injection of 10^6^ MSCs is a commonly used dosage in similar studies. Numerous publications have employed this dosage (e.g., PMID: 22752635, PMID: 33757765, PMID: 31133062). In the revised manuscript, we have included the baseline body weight of the mice (approximately 22-25 grams per mouse) before the experiments. The gender of the animals (female) was clearly stated in the original manuscript. Regarding the amount of hUC-MSCs homing to the liver, we did not specifically quantify the exact cell number. However, we generated GFP-labeled hUC-MSCs and injected them into mice under different treatment conditions. As shown in the revised Figure S2, hUC-MSCs were able to reach and be retained in the liver, with higher levels observed in injured liver tissues. We believe these results provide sufficient evidence to support our conclusion.

**Stastics: "GraphPad Prism8" is application name, please provide what kind of statsitic was used in each experiment.**

Response: The methods for statistical analyses had been clearly described in the figure legends.

**Figue2: since upper and lower panel did not match, it is confusing to understand. In panel B, what yellow dots and white dots are indicating.**

Response: Thank you for your comment. In the revised Figure 2, we have added dashed boxes in parts A and B to clearly indicate the correspondence between the upper and lower panels. The lower panels present magnified views of the specific regions highlighted in the upper panels. Yellow dots mark TUNEL-positive cells. The white areas are likely due to exceptionally strong apoptotic fluorescence signals from individual cells.

**Reviewer #2: the manuscript presents a comprehensive and well-structured investigation into the therapeutic potential of human umbilical cord-derived mesenchymal stem cells (hUC-MSCs) in autoimmune hepatitis (AIH), particularly focusing on the suppression of ferroptosis as a key protective mechanism**

**1. Abstract Revision – Improve Specificity and Flow**

**Current Issue: The abstract is informative but lacks precision in quantitative terms and slightly overstates mechanistic conclusions.**

Response: Thank you for your comment. We have revised the Abstract to enhance its conciseness and precision, ensuring that it now more accurately reflects the key findings and perspectives of our study. We appreciate your feedback, which has been invaluable in improving the clarity of our manuscript.

**2. Sample Size Limitation, Only n = 3 per group in key experiments.**

**While preliminary data is still valuable, such a small number limits statistical power and generalizability. A sample size of 3 severely underpowers the study and affects statistical reliability.**

Response: Thank you for your comment. We have repeated the experiments using six additional mice and incorporated the new results into the revised figures.

**3. Mechanistic Causality Needs Clarification**

**While ferroptosis marker modulation is demonstrated, causal inference remains correlative. so the paper lack of Causal Validation of Ferroptosis Pathway**

Response: We understand the reviewer’s concern and have acknowledged this limitation in the Discussion section. Indeed, the exploration of ferroptosis-related mechanisms in this study remains relatively preliminary. While our current work primarily demonstrates an association between MSC-based therapy and ferroptosis inhibition, a more in-depth investigation into the underlying mechanisms will be pursued in a separate project using a broader range of experimental approaches. To further address this issue in the present study, we plan to conduct additional rescue experiments, such as the application of ferroptosis agonists, to clarify the causal relationship between the protective effects of MSCs and ferroptosis inhibition. We will publish these data in the future.

**4. Missing Controls: A ConA + vehicle control group (without MSCs) for comparison in qPCR and Western blot analysis should be explicitly stated and shown.**

Response: We suspended the MSCs in PBS and administered the same volume of PBS, used for MSC suspension, to the mice in the ConA group, serving as the appropriate control as suggested by the reviewer. This information has been included in the revised manuscript.

**5. There is minimal detail on how hUC-MSCs were characterized for identity, purity, and viability before administration.**

Response: We apologize for the missing details and have included them in the revised manuscript and Figure S1.

**6. Clarify Timing of Sampling Post-ConA. Inconsistent Statements: “24h after ConA injection” is mentioned in methods, but survival data are observed up to 18h.**

Response: The survival data (Figure A) show that all mice in the ConA group died within 18 hours, whereas all mice in the other groups survived for at least 24 hours, as indicated by the red line at the top.
