# Supplementary material for: Human umbilical cord-derived mesenchymal stem cells alleviate autoimmune hepatitis by inhibiting hepatic ferroptosis
Source: PLoS One. 2025 Dec 4;20(12):e0337060. doi: 10.1371/journal.pone.0337060 (PMC12677442; doi:10.1371/journal.pone.0337060)
Supplement: Attachment — Submitted filename: Response to Reviewers.docx [file pone.0337060.s008.docx]

Journal Requirements:

1. If the reviewer comments include a recommendation to cite specific previously published works, please review and evaluate these publications to determine whether they are relevant and should be cited. There is no requirement to cite these works unless the editor has indicated otherwise. 

2. Please review your reference list to ensure that it is complete and correct. If you have cited papers that have been retracted, please include the rationale for doing so in the manuscript text, or remove these references and replace them with relevant current references. Any changes to the reference list should be mentioned in the rebuttal letter that accompanies your revised manuscript. If you need to cite a retracted article, indicate the article’s retracted status in the References list and also include a citation and full reference for the retraction notice.

3. Please provide the original blot images for Figure 2S.
Response: Thanks for the comment. We have provided the original blot image for panel C of Figure S2 in the separate file "uncropped western blot-2". Please note that this is the only panel from this figure S2 for which a new original blot is required.

4. Please clarify the reasons for the authorship changes and specify why the changes were not made at initial submission.

Response: Thanks for the comment.During the revision process, we have added Mr. Luoshi Zhang as a co-author to the manuscript. His substantial contributions to performing the critical experiments outlined in the response letter and his analysis of the new data fully meet the criteria for authorship.

Additional Editor Comments (if provided):

It is a good paper overall, but there are a few technical points I have raised.
 **The abstract mentions “metabolomic analysis revealed modulation of pathways related to ferroptosis,” but does not specify whether pathway enrichment or specific metabolites were validated. A clearer statement of the level of evidence (correlation vs. validation) would strengthen accuracy.**
Response: Thanks for the advice. We have rephrased the sentence as follows: “Metabolomic and ontology analyses of mouse liver tissue samples revealed that hUC-MSCs treatment altered the levels of metabolites (Glu derivatives and peptides) functionally associated with ferroptosis-related pathways.” Since we didn’t perform validation, emphasizing ontology analysis should be sufficient to clarify this.

**The abstract implies that hUC-MSCs inhibit ferroptosis as the mechanistic basis of protection (“through inhibiting the ferroptosis pathway”).
Unless ferroptosis inhibition was directly tested (e.g., by using ferroptosis activators/inhibitors or rescue assays), it would be more accurate to phrase this as: “...potentially through modulation of ferroptosis-related pathways.”
“hUC-MSCs” defined twice — keep only once**
Response: We appreciate the advice. The relevant text has been modified accordingly. Yet, we can’t find a duplicated definition of hUC-MSCs in the Abstract. Following your suggestion, we have removed the redundant definition of hUC-MSCs from the Introduction, while retaining the single definition in the Abstract.

**The ConA model indeed induces T-cell–mediated immune hepatitis, but calling it “autoimmune hepatitis” should be qualified — it is an AIH-like murine model, not true autoimmune hepatitis .. please correct this**

Response: Thanks for the advice. We have made the modification in the revised manuscript. As suggested, we have changed "ConA-induced AIH mouse model" to "ConA-induced AIH-like mouse model" throughout the manuscript.
